# Engineering and Evolution of Molecular Chaperones and Protein Disaggregases with Enhanced Activity

**DOI:** 10.3389/fmolb.2016.00008

**Published:** 2016-03-15

**Authors:** Korrie L. Mack, James Shorter

**Affiliations:** ^1^Department of Biochemistry and Biophysics, Perelman School of Medicine at the University of PennsylvaniaPhiladelphia, PA, USA; ^2^Biochemistry and Molecular Biophysics Graduate Group, Perelman School of Medicine at the University of PennsylvaniaPhiladelphia, PA, USA

**Keywords:** chaperone, disaggregase, engineering, evolution, Hsp70, GroEL, SPY, Hsp104

## Abstract

Cells have evolved a sophisticated proteostasis network to ensure that proteins acquire and retain their native structure and function. Critical components of this network include molecular chaperones and protein disaggregases, which function to prevent and reverse deleterious protein misfolding. Nevertheless, proteostasis networks have limits, which when exceeded can have fatal consequences as in various neurodegenerative disorders, including Parkinson's disease and amyotrophic lateral sclerosis. A promising strategy is to engineer proteostasis networks to counter challenges presented by specific diseases or specific proteins. Here, we review efforts to enhance the activity of individual molecular chaperones or protein disaggregases via engineering and directed evolution. Remarkably, enhanced global activity or altered substrate specificity of various molecular chaperones, including GroEL, Hsp70, ClpX, and Spy, can be achieved by minor changes in primary sequence and often a single missense mutation. Likewise, small changes in the primary sequence of Hsp104 yield potentiated protein disaggregases that reverse the aggregation and buffer toxicity of various neurodegenerative disease proteins, including α-synuclein, TDP-43, and FUS. Collectively, these advances have revealed key mechanistic and functional insights into chaperone and disaggregase biology. They also suggest that enhanced chaperones and disaggregases could have important applications in treating human disease as well as in the purification of valuable proteins in the pharmaceutical sector.

## Introduction

The advancement of protein engineering has enriched our understanding of protein structure, function, and folding. Protein-engineering endeavors have met both challenge and opportunity in designing proteins with desirable or novel functions. An exciting assortment of approaches has been developed to optimize starting protein scaffolds, ranging from the development of site-directed mutagenesis in the early 1980s (Dalbadie-Mcfarland et al., 1982; Sigal et al., 1982; Winter et al., 1982), to more recent computational design efforts (Jiang et al., 2008; Siegel et al., 2010). A wide spectrum of proteins have been successfully engineered and evolved to adopt new or improved function. From engineering DNA polymerases to better recognize unnatural nucleotides (Laos et al., 2014), to introducing catalytic activity into non-enzymatic scaffolds (Korendovych et al., 2011), protein engineering has enhanced our repertoire of knowledge far beyond solely a fundamental understanding of protein structure and function.

Despite such progress, there are major challenges to protein engineering, mostly rooted in the abundance of constraints embedded in natural protein sequences and structures (Dutton and Moser, 2011). Understanding the basis for these constraints has proven challenging, as evolution has sculpted epistatic relationships between residues and structural domains in proteins that are necessary for thermodynamic stability, dynamics, allostery, or complex assembly (Liberles et al., 2012; Gong and Bloom, 2014; Perica et al., 2014; Sikosek and Chan, 2014). Amino acids within natural proteins may display Müllerian interdependency and consequently may have accumulated irreversible complexity (Dutton and Moser, 2011). Moreover, natural proteins may also have been subjected to multiple, simultaneous and diverse selection pressures leading to complexity from Darwin's principle of multiple utility (Dutton and Moser, 2011). These various historical constraints and complexities can confound engineering approaches. Indeed, historical contingency and entrenchment due to epistasis play a critical role in protein evolution (Weinreich et al., 2006; Bridgham et al., 2009; Bloom et al., 2010; Gong et al., 2013; Harms and Thornton, 2014; Shah et al., 2015). For example, the evolutionary trajectory of influenza nucleoprotein revealed that stabilizing substitutions were essential in compensating for otherwise intolerable destabilizing changes that were beneficial to the protein in an adaptive sense (Gong et al., 2013). Thus, epistatic relationships between residues pose a large challenge to protein engineers who seek to achieve more than a modest improvement in protein activity. Consequently, some groups have steered away from utilizing natural protein scaffolds as a starting point for engineering, and instead create completely artificial, human-made proteins (Lombardi et al., 2000; Faiella et al., 2009; Anderson et al., 2014; Solomon et al., 2014). Nonetheless, we are still in the very early phases of designing artificial proteins for specific functional purposes. Moreover, the challenges inherent to engineering natural proteins are not insurmountable and the extent to which epistasis as well as Müllerian and Darwinian complexity might restrict engineering is poorly defined. Indeed, surprisingly large and utilitarian gains in activity can be achieved with remarkably small changes to natural proteins (Wang et al., 2002; Aponte et al., 2010; Jackrel et al., 2014; Quan et al., 2014).

It is important to recognize that natural proteins are not perfect. Moreover, it is not always clear where natural proteins lie in terms of the adaptive landscape. For example, it is not known what proportion of natural proteins resides at local adaptive optima with room for substantial improvement. Indeed, natural proteins are likely only good enough to get an organism to reproductive age. Natural selection acts less powerfully on genetic variation expressed at post-reproductive age, and thus many proteins likely harbor “late-expressing” harmful mutations (Medawar, 1952). Some of these mutations may predispose specific proteins to deleterious misfolding in the aging individual, as occurs in various fatal neurodegenerative diseases such as Alzheimer's disease (AD), Parkinson's disease (PD), Huntington's disease (HD), frontotemporal dementia (FTD), and amyotrophic lateral sclerosis (ALS), in which protein misfolding causes selective neuronal degeneration (Cushman et al., 2010). Moreover, natural molecular chaperones or proteostasis networks (Balch et al., 2008) in general may be poorly equipped to counter deleterious protein misfolding in the context of aging individuals due to a lack of selection for buffering protein misfolding under these circumstances, which occur long after reproductive age. Emerging evidence suggests that the proteostasis network declines in the soma at reproductive age due to conflict between the germ line and soma (Labbadia and Morimoto, 2014, 2015a,b). Defining effective strategies to remediate the toxic protein misfolding underlying several age-related neurodegenerative diseases is of great importance. Thus, we are particularly interested in harnessing the power of protein engineering against detrimental protein misfolding events. Specifically, we would like to engineer molecular chaperones or protein disaggregases with potentiated activity, which might be particularly useful in the specific environment of declining proteostasis in aging individuals.

In the context of the extremely crowded cellular environment, the folding of polypeptide chains into precise functional structures is a daunting task (van Den Berg et al., 1999; Uversky et al., 2002; Sarkar et al., 2013). Upon stress, the proteostasis network may become compromised, and proteins may evade the various safeguards against improper protein folding and ultimately aggregate. Molecular chaperones are key players in the maintenance of proper protein folding and overall proteostasis. Chaperones are required by newly synthesized proteins to ensure both accurate folding and to prevent aggregation. Indeed, chaperones function both cotranslationally and in times of cellular stress (Hartl and Hayer-Hartl, 2002). Chaperones are also involved in triaging misfolded proteins for degradation and the trafficking of proteins (Hartl et al., 2011). Although molecular chaperones are diverse in terms of structure and mechanism, they generally identify unstructured or hydrophobic stretches on their respective client substrates that are inappropriately exposed in misfolded conformations (Bukau et al., 2000; Hartl and Hayer-Hartl, 2002). However, variations on this theme abound. For example, the eukaryotic chaperonin, TRiC, recognizes polar and nonpolar residues in substrates via a combinatorial mechanism (Joachimiak et al., 2014). Most chaperones foster effective folding in an ATP-dependent fashion, where substrate affinity is regulated by the binding and release of ATP (Hartl and Hayer-Hartl, 2002). Despite sharing this ATP-driven refolding activity, different classes of molecular chaperones possess distinct refolding mechanisms, whereby some release unfolded substrates to be refolded, and others form a protective cage around the substrate, allowing for refolding (Hartl and Hayer-Hartl, 2002). Molecular chaperones as a collective are essential for all life as spontaneous protein folding in itself is not sufficient (Hartl, 2011; Horwich, 2011; Rothman and Schekman, 2011). For example, one type of molecular chaperone, Hsp70, is essential for all eubacteria and eukaryotes, but is bafflingly absent from the majority of archaebacteria (Large et al., 2009; Powers and Balch, 2013). Hsp60 appears to be essential for almost all life and so far has been found in every eukaryotic and prokaryotic organism with the curious exception of *Mycoplasma* species (Henderson et al., 2013; Powers and Balch, 2013). These pathogenic eubacteria have very small genomes and presumably have been able to dispense with Hsp60 via increased reliance upon Hsp70 or via evolution of Hsp60 clients to fold propitiously without Hsp60 (Fujiwara et al., 2010; Powers and Balch, 2013; Georgescauld et al., 2014; Ishimoto et al., 2014). Typically, as genomes and proteomes expand during evolution so does the number of representatives from the canonical Hsp90, Hsp70, Hsp60, Hsp40, and small heat shock protein families (Powers and Balch, 2013). Undoubtedly, molecular chaperones are influential modulators of proteostasis, and are indispensable for efficacious folding of proteins into their native structures *in vivo*.

One solution to combat detrimental protein misfolding and aggregation might be to create a completely artificial molecular chaperone or protein disaggregase *de novo*. This grand challenge has not yet been met, but warrants investigation. We are interested in tailoring existing chaperones and disaggregases to better refold client substrates. Evolved molecular chaperones with highly enhanced refolding or aggregation prevention ability could prove advantageous in targeting misfolded proteins relevant to neurodegenerative disease. Here, we review several important examples of chaperone engineering, which highlight essential themes in successfully developing enhanced chaperone variants.

## An unbiased approach to engineering GroEL

**Figure 1:**
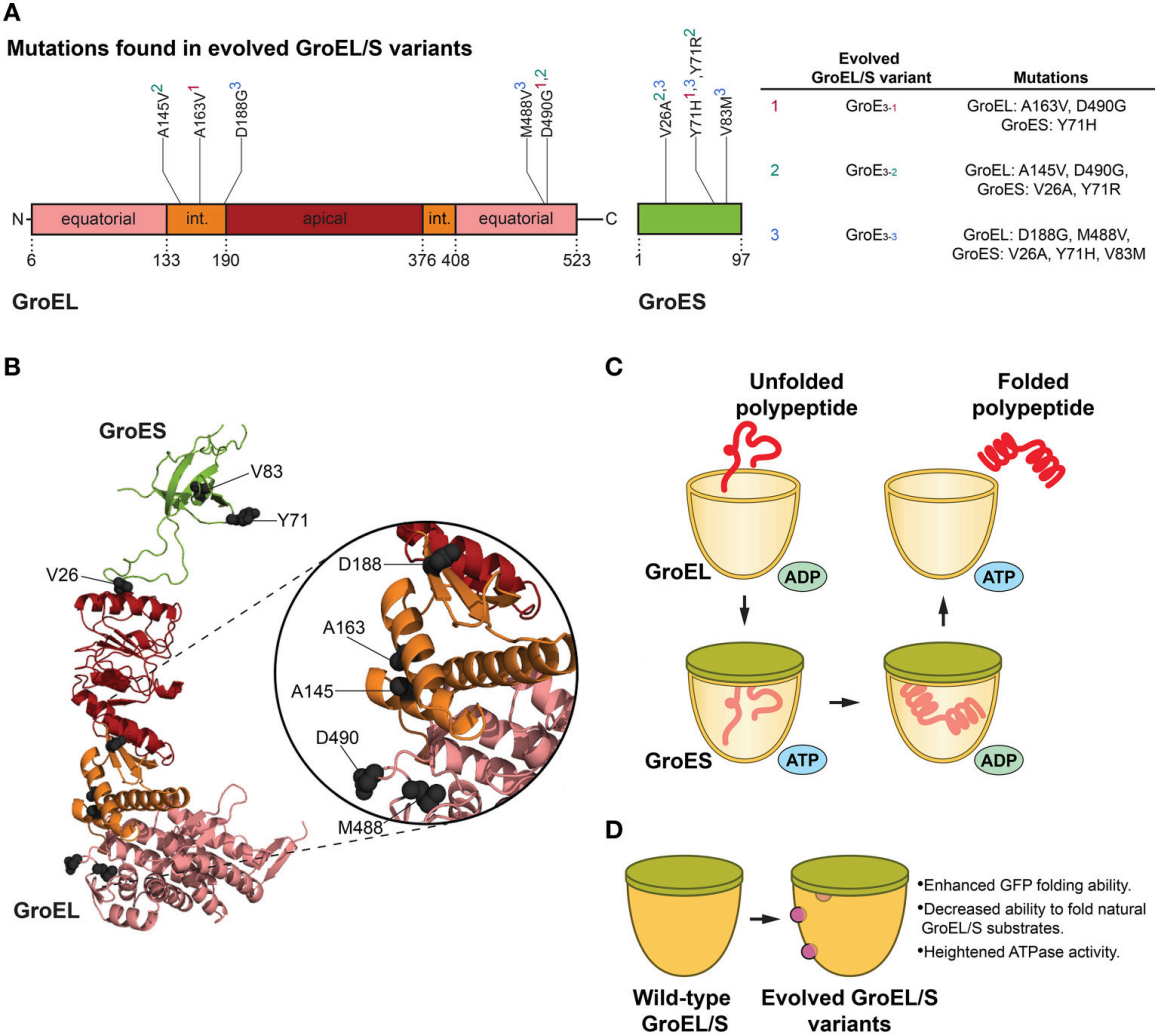
**GroEL/S protein folding cycle and features of evolved variants. (A)** Mutations in evolved GroEL/S variants surface in the first intermediate domain (int.) and second equatorial domain of GroEL, and throughout GroES (Wang et al., 2002). Interestingly, no mutations arise in the apical substrate-binding region (Wang et al., 2002). Superscripts in the table (based on Table 1 in Wang et al., 2002) correspond to the GroEL/S variants isolated in the final round of evolution (e.g., A163V, D490G, and Y71H are found in the GroE_3−1_ variant). **(B)** Structure (PBD ID: 1AON) of a GroEL/S subunit in the *cis*-ring of a GroEL/S-ADP complex (Xu et al., 1997) showing the positions of mutations (gray spheres) in evolved GroEL/S variants. Colors correspond to **(A)**. Based on Figure 5B in Wang et al. (2002). **(C)** GroEL/S protein folding cycle. Substrate binds to GroEL, and upon binding of ATP and GroES to the same ring, substrate is moved into the chamber. The substrate is folded in the protective cage environment (the exact mechanism is still debated), and is released upon ATP hydrolysis and dissociation of GroES (Hendrick and Hartl, 1995; Hartl et al., 2011). Only one heptameric GroEL ring is shown for simplicity. **(D)** Features of evolved GroEL/S variants. Variants isolated from the final round of directed evolution show enhanced GFP-folding ability, decreased ability to fold natural GroEL/S substrates, and heightened ATPase activity (Wang et al., 2002).

In the early 2000s, Weissman and colleagues successfully engineered enhanced variants of the Hsp60 molecular chaperone, GroEL (Wang et al., 2002). GroEL is a member of the group I chaperonins which are found in bacteria, chloroplasts, and mitochondria (Henderson et al., 2013; Gupta et al., 2014). Group I chaperonins are comprised of heptameric rings, and interact with co-chaperone partners to capture substrate in a protective folding cage (Henderson et al., 2013; Gupta et al., 2014). GroEL is found in a large number of bacteria, and together with its co-chaperone GroES is extremely well characterized (Castanié-Cornet et al., 2014). It is estimated that ~10% of cytosolic proteins in *E. coli* interact with GroEL, as it provides an environment to hinder aggregation of its substrates, and favor their folding (Clare and Saibil, 2013). Structurally, GroEL is composed of two back-to-back heptameric rings, in which each subunit of the heptamer is comprised of three types of domains (Clare and Saibil, 2013). Intermediate domains connect the central apical domain, responsible for binding GroES and substrates, to the two equatorial domains, which bind ATP and facilitate interactions between and within subunits (Figures 1A,B; Saibil et al., 2013). A hydrophobic area in the apical domain binds substrate polypeptides ranging in size from ~20 to 60 kDa, which can then be confined to the protective folding chamber that is sealed by a GroES lid (Castanié-Cornet et al., 2014; Hayer-Hartl et al., 2016). GroEL can also promote folding of proteins too large to be encapsulated in the chamber (Chaudhuri et al., 2009; Hayer-Hartl et al., 2016). The exact mechanism by which GroEL provides folding assistance to substrates is still debated and range from GroEL providing a passive box for aggregation prevention, to actively changing substrate folding trajectory, to folding through forced unfolding (Figure 1C; Hendrick and Hartl, 1995; Shtilerman et al., 1999; Thirumalai and Lorimer, 2001; Horst et al., 2007; Apetri and Horwich, 2008; Lin et al., 2008; Priya et al., 2013; Saibil et al., 2013; Yang et al., 2013; Fei et al., 2014; Gupta et al., 2014). Regardless, the allosteric communication between the two GroEL rings effectively promotes polypeptide folding, ATP hydrolysis, and subsequent release of the polypeptide (Jewett and Shea, 2010).

Since GroEL is a thoroughly studied allosteric machine, it is a well-suited target for chaperone engineering. Using a directed evolution approach, Weissman and colleagues isolated variants of GroEL and co-chaperone GroES that showed enhanced ability to fold green fluorescent protein (GFP; Wang et al., 2002). GFP was an advantageous substrate for this study in that folding can be readily followed by monitoring GFP fluorescence in cells (Wang et al., 2002). Directed evolution comprised of rounds of *in vivo* screening coupled with *in vitro* DNA shuffling was used to attain GroEL/S variants with up to ~8-fold improvement in GFP folding activity (Figures 1A,B,D). The initial round of evolution consisted of site-directed mutagenesis in the substrate-binding region of the chaperonin, as well as random mutagenesis in the GroEL/S operon (Wang et al., 2002). The library generated from this round was transformed into GFP-expressing *E. coli* cells, and the brightest-glowing colonies were selected for a second round of evolution comprised of DNA shuffling (Wang et al., 2002). The last cycle of evolution yielded several enhanced variants with modifications to both components of the GroEL/S machine (Wang et al., 2002). Importantly, it was determined that the improvement in folding ability was not reliant on other molecular chaperones in the cell, and was solely a function of the changes to the GroEL/S machine (Wang et al., 2002).

In evolving proteins to generate improved variants, it is critical to determine whether the observed enhancement is the result of a broad improvement in activity or increased specificity toward the substrate used in directed evolution. This distinction offers insight into the specialization of chaperones for particular substrates, or their inherent generality toward a wide breadth of substrates. To this end, Weissman and colleagues tested the ability of their improved variants to fold natural GroEL/S substrates. The variants were not effective at folding the substrates tested, with the exception of one variant (GroE_3−1_, see Figure 1A) which could successfully fold mitochondrial rhodanese (Wang et al., 2002). Interestingly, these results indicated that the gain in GFP folding activity was derived from a change in substrate specificity from endogenous substrates to unnatural GFP, and demonstrated that the enhancement of GFP folding activity manifested as a trade-off that reduced the ability of GroEL/S to fold its natural substrates (Wang et al., 2002).

Utilizing a directed evolution approach makes it possible to uncover interesting chaperone variants in an unbiased fashion, which is a major strength in terms of fully exploring sequence space and discovering activity-enhancing mutations that may be less intuitive. Remarkably, by the final cycle of directed evolution, mutations in the most improved GroEL/S variants were not found in the substrate-binding domain of the chaperonin (despite this region being used for random mutagenesis), but rather in the N-terminal intermediate domain or near the nucleotide-binding region (Wang et al., 2002). Consistent with this finding is work by Kawe and Plückthun, whereby an alternate strategy to modify the substrate-binding region in the apical domain of GroEL was employed (Kawe and Plükthun, 2006). To alter substrate specificity, a library of GroEL variants was created through randomization of residues throughout the apical domain that were thought to directly interact with clients (Kawe and Plükthun, 2006). The library was screened *in vivo* where tuned GroEL variants were selected, then more thoroughly scrutinized *in vitro* (Kawe and Plükthun, 2006). Interestingly, no variants with dramatically improved refolding activity were isolated from engineering solely the substrate-binding region (Kawe and Plükthun, 2006). The results from this approach highlight the difficulties of directly targeting the substrate-binding domain for enhancement of chaperone activity (Kawe and Plükthun, 2006). Kawe and Plückthun suggest a “narrow mutational window” in the apical domain, since in addition to binding substrate it must also bind GroES, which is critical in the allosteric regulation of the GroEL system (Kawe and Plükthun, 2006). Indeed, these findings illustrate the difficulty of engineering natural proteins due to constraints imposed by Darwin's principle of multiple utility.

Mutations that enhanced GroEL activity increased the ATPase activity of GroEL, and improved the ability of GroES to inhibit GroEL ATPase activity (Figures 1A,B; Wang et al., 2002). Additionally, studying a GroES variant that surfaced from the screening showed that increasing the overall polarity of the GroEL/S folding chamber is important for improved GFP folding activity (Wang et al., 2002). Thus, a delicate balance between altering the surface properties of the GroEL/S chamber and the GroEL ATPase cycle was crucial for optimized activity.

When the GFP folding activity of the GroE_3−1_ variant was tested *in vitro*, the improvement was surprisingly modest compared to the wild-type chaperonin (Wang et al., 2002). This result supports the idea that the improved GroEL/S variants are not necessarily “hyperactivated,” and are actually enhanced *in vivo* because they are able to “ignore” other substrates and more effectively focus on GFP. Thus, this unbiased approach yielded GFP-specific GroEL/S variants with a contracted substrate repertoire as opposed to a generally enhanced chaperonin (Wang et al., 2002), indicating that the generalist GroEL/S can be tuned to recognize only specific substrates. Engineering the GroEL/S chaperonin highlights a fascinating theme in chaperone engineering: very slight modifications (here, only two missense mutations to GroEL, and one to GroES, were required for optimized activity) to chaperone sequence can translate to highly improved folding machines for specific substrates. GroEL/S has likely evolved to be a generalist to assist the folding of diverse polypeptides and also functions as a capacitor to buffer genetic variation and enable evolvability of client proteins (Fares et al., 2002; Tokuriki and Tawfik, 2009). However, this generalist activity inescapably incurs a cost of reduced activity against a specific subset of clients. Thus, GroEL and other molecular chaperones likely exhibit an unavoidable functional trade off for some substrates akin to the proverbial jack-of-all-trades, master of none. This intrinsic and likely historical constraint suggests that chaperone activity can often be evolved toward specific, dedicated substrates if necessary, which could have important implications for specifically targeting proteins that misfold in neurodegenerative disease.

Interestingly, in eukaryotes the group II chaperonin (group II chaperonins contain a “built-in” lid and do not require a GroES-type cochaperone for activity), TRiC, evolved to become hetero-oligomeric as opposed to homo-oligomeric GroEL (Lopez et al., 2015). Thus, TRiC contains eight different subunits per ring (Lopez et al., 2015). Each subunit has a substrate-binding site, which specifies recognition of distinct substrate motifs encompassing both polar and nonpolar determinants (Joachimiak et al., 2014). This subunit diversification enables a combinatorial mode of substrate binding capable of recognizing a large and complex sequence space, which might even have empowered the expansion of eukaryotic proteomes to acquire essential proteins with novel folds, complex topologies, and functions (Yam et al., 2008; Joachimiak et al., 2014). Indeed, many essential eukaryotic proteins, e.g., actin and Cdc20, can only be folded in a TRiC-dependent manner. Moreover, TRiC is a powerful inhibitor of misfolding of mutant huntingtin exon 1 containing an expanded polyglutamine tract, which is connected with HD (Kitamura et al., 2006; Tam et al., 2009; Shahmoradian et al., 2013; Sontag et al., 2013). Here too, we wonder if TRiC activity is limited by functional trade-offs as with GroEL and GFP (Wang et al., 2002). Clearly, TRiC ultimately fails to prevent misfolding of polyglutamine-expanded huntingtin in HD. Thus, perhaps TRiC could be engineered or evolved to possess enhanced activity against huntingtin or other proteins connected with neurodegeneration. One interesting strategy is to harness and perhaps even tailor the substrate-binding apical domain of TRiC subunit CCT1, ApiCCT1, which can suffice to antagonize polyglutamine-expanded huntingtin exon 1 misfolding (Sontag et al., 2013). Despite the difficulty in finding mutations in the apical domain of GroEL that enhance activity (Wang et al., 2002; Kawe and Plükthun, 2006), whether the same is true for TriC remains unclear. TriC operates without a GroES analog (Lopez et al., 2015), and so the constraint of maintaining GroES binding is alleviated in the context of TriC engineering. ApiCCT1 demonstrates a remarkable ability to enter cells due to some sequence similarity with the HIV Tat protein cell-transduction domain (Sontag et al., 2013). ApiCCT1 delivered exogenously in this way can exert therapeutic effects in HD cell models (Sontag et al., 2013).

## Flexibility enhances activity in Spy, an ATP-independent chaperone

**Figure 2:**
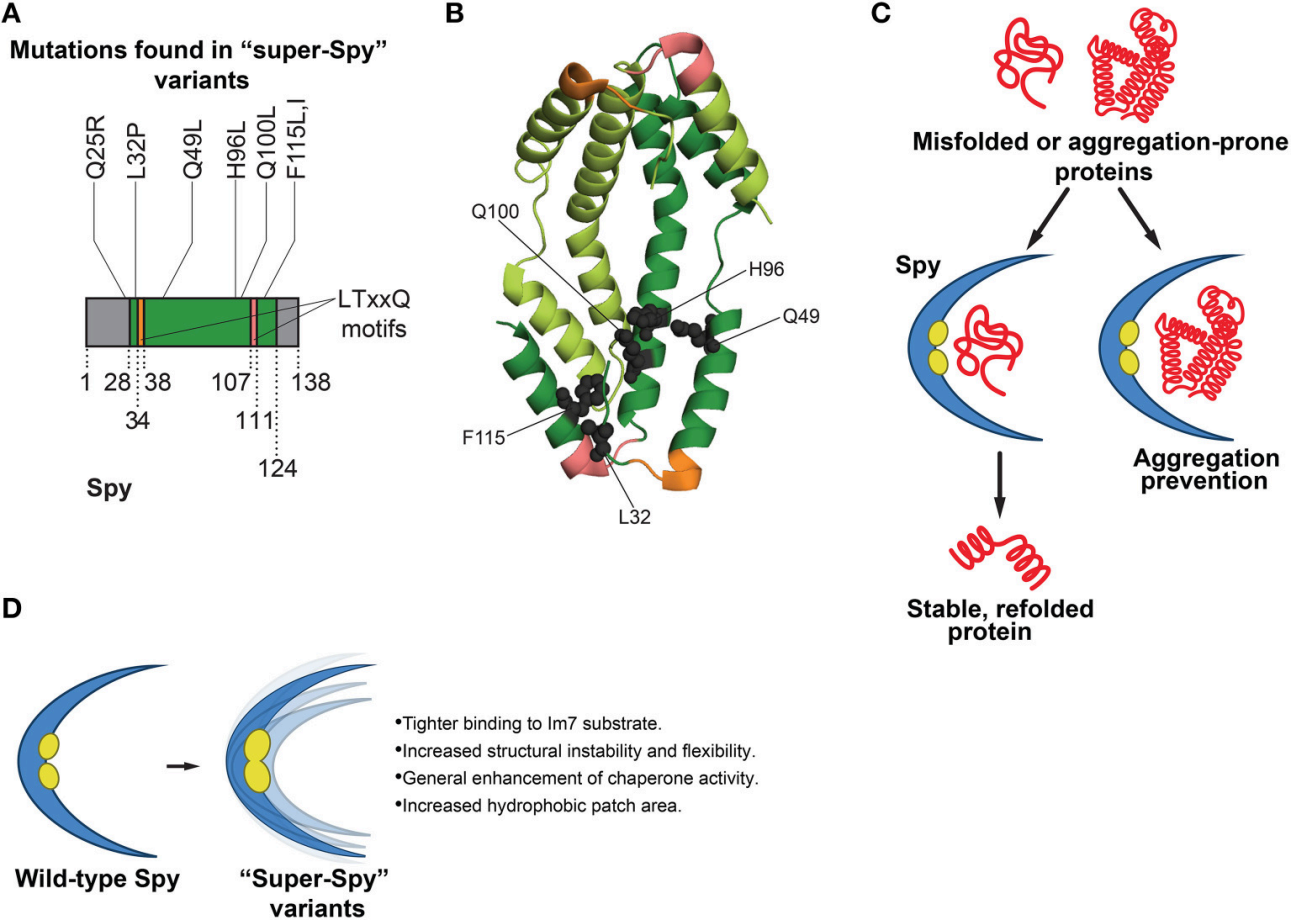
**Functions of Spy in protein folding and features of super-Spy variants. (A)** Most mutations in super-Spy variants result in a change in residue character to hydrophobic (Quan et al., 2014). Of the initial 65 isolated Spy variants, 74% contained the Q100L mutation (Quan et al., 2014). Residues are numbered according to the mature protein region of Spy. Gray regions denote residues not pictured in **(B)** structure (this includes the Q25 position). **(B)** Structure (PDB ID: 3O39) of a Spy dimer (Quan et al., 2011) showing the positions of mutations (gray spheres) in super-Spy variants. One Spy subunit is shown in dark green, and the other in light green. Mutations are shown for only one subunit, and LTxxQ motifs (Kwon et al., 2010) are shown for both subunits. Twenty-eight N-terminal residues and 14 C-terminal residues are not pictured as they are disordered (Quan et al., 2011). Colors correspond to **(A)**. **(C)** Functions of Spy in protein folding. Spy aids in refolding of substrates independently of ATP. Substrate can fully fold while bound to Spy, or Spy can prevent aggregation by holding substrate in a protective, cradle-shaped environment (Quan et al., 2011, 2014; Stull et al., 2016). **(D)** Features of super-Spy variants. Super-Spy variants show tighter binding to the Im7 substrate, increased structural instability and flexibility, general enhancement of chaperone activity, and an increased hydrophobic patch area (Quan et al., 2014).

Many molecular chaperones, such as GroEL, require ATP to function in protein folding. An interesting deviation from this ATP dependence is the chaperone Spy, which contains two LTxxQ motifs and forms a cradle-shaped dimer (Figures 2A,B; Kwon et al., 2010; Quan et al., 2011). Spy was isolated in *E. coli* by Bardwell and colleagues in 2011 (Quan et al., 2011), and its ATP independence is similar to specific molecular chaperones in mammals located in the ATP-scarce extracellular space (Wyatt et al., 2013). In search of *E. coli* variants that promote protein stability and folding in the periplasm, Bardwell and colleagues unearthed a chaperone that stands in its own class in terms of both structure and function. The basis for the genetic system used to identify Spy is interesting in that it couples protein folding and stability to selectable markers, ultimately yielding quantitative information regarding *in vivo* protein stability (Quan et al., 2011). The system is comprised of a fusion between β-lactamase, necessary for resistance to penicillin V, and DsbA, involved in resistance to cadmium (Quan et al., 2011). A highly unstable form of immunity protein 7 (Im7) was inserted between these two selection markers, allowing for the inherent stability of Im7 to be paired with the penicillin and cadmium resistance of the *E. coli* strain (Quan et al., 2011). *E. coli* isolates that demonstrated an increase in penicillin resistance were then screened for improved cadmium resistance (Quan et al., 2011). More generally, an enhancement in protein stability translated to increased resistance to the selection markers, providing powerful selection criteria to discover new chaperones in the periplasm, which is devoid of ATP (Quan et al., 2011). In *E. coli* isolates that improved the folding of, and thereby increased expression of Im7, Spy was incredibly overexpressed, constituting almost half of the protein content of the periplasm (Quan et al., 2011). Bardwell and colleagues then demonstrated that an increase in Spy levels is sufficient to enhance Im7 levels, and that Spy served as a proficient chaperone in terms of helping to refold soluble, misfolded proteins and halting protein aggregation in an *in vitro* setting (Quan et al., 2011). The mechanism of Spy-mediated client folding was recently investigated to determine whether substrate could fold while bound to Spy, or if it must be released for folding to ensue (Stull et al., 2016). Interestingly, Spy is capable of binding with similar affinities the native, intermediate and unfolded states of its substrate Im7 (Stull et al., 2016). As such, Im7 can fully fold while bound to Spy, and Spy does not need to release Im7 for folding to occur (Figure 2C; Stull et al., 2016). This outcome is fascinating in the greater context of ATP-independent chaperones, as it suggests a more dynamic role for these chaperones in substrate folding beyond mere “holdases” that prevent aggregation.

To engineer enhanced Spy variants, a “stability biosensor” was employed, comprised of the original unstable Im7 variant that Spy was shown to stabilize (Quan et al., 2011) directly inserted into β-lactamase (Quan et al., 2014). Error-prone PCR was used to create a large plasmid library of Spy variants (Quan et al., 2014). The library was transformed into an *E. coli* strain that lacked Spy, yet contained the biosensor (Quan et al., 2014). Spy expression was induced by IPTG. Thus, increased IPTG levels would translate to greater Spy levels and enhanced resistance to penicillin, with the underlying premise that activity-improving mutations in Spy would lead to greater penicillin resistance (Quan et al., 2014). Sixty-five Spy variants that displayed enhanced penicillin resistance were isolated (Quan et al., 2014). Interestingly, about three-quarters of the Spy variants surfacing from the selection possessed a Q100L mutation, while the others contained a variety of mutations (Figures 2A,B; Quan et al., 2014). It was established that the enhancement in antibiotic resistance was not a result of a more general mechanism such as increased Spy expression, as all of the Spy variants were present at levels similar to that of wild-type Spy (Quan et al., 2014). *E. coli* strains expressing enhanced Spy variants demonstrated higher minimal inhibitory concentrations than the strain expressing wild-type Spy, signifying that the isolated Spy variants possessed greater activity than the wild-type protein (Quan et al., 2014). Although one might expect the Spy variants to be deficient in folding other substrates besides Im7 (since this protein was used in the genetic selection), the variants surprisingly showed a general enhancement in chaperone activity toward other substrates (Figure 2D; Quan et al., 2014). Thus, the Spy variants are distinct from the GroEL variants isolated by Weissman and colleagues (Wang et al., 2002), which traded general GroEL function for improved GFP folding activity. The general chaperone activity of purified Spy variants was tested *in vitro* against chemically denatured aldolase, as well as reduced denatured α-lactalbumin (Quan et al., 2014). The variants were termed “super-Spy” as they displayed enhanced aggregation prevention of these chaperone substrates, and were more active in aldolase refolding (Figure 2D; Quan et al., 2014).

Spy is a structurally unique chaperone in that it forms a dimer with a novel cradle-shape, with one very concave surface, and one very convex surface (Figure 2B; Kwon et al., 2010). The concave portion of Spy contains hydrophobic patches believed to be important for substrate binding (Kwon et al., 2010; Quan et al., 2011). Spy is an incredibly thin protein, with an average cross-section thickness of the cradle of 9.2 Å (Quan et al., 2011). Mapping of the individual mutations to the crystal structure of Spy revealed that most of the super-Spy mutations clustered together, with the majority residing next to the two major hydrophobic patches (Figures 2B,C; Quan et al., 2014). Three mutations including the most common, Q100L, resulted in a change in residue character from charged or polar to hydrophobic, which ultimately expanded the hydrophobic surface area (Figure 2D; Quan et al., 2014). Did these mutations enhance Spy chaperone activity via increased affinity for substrate proteins, or through an allosteric mechanism? Bardwell and colleagues probed the binding of Im7 to Spy through hydrogen-deuterium exchange, a limited proteolysis approach, and crosslinking experiments (Quan et al., 2014). Hydrogen-deuterium exchange studies and limited proteolysis indicated that large areas of Spy engage Im7 (Quan et al., 2014). Moreover, the concave surface of Spy served as the major Im7 binding site (Quan et al., 2014). Super-Spy variants displayed tighter binding to substrates (Figure 2D; Quan et al., 2014). Interestingly, a negative correlation between super-Spy variant activity *in vivo* and thermodynamic stability *in vitro* was found, indicating the less stable Spy variants displayed improved chaperone activity (Quan et al., 2014). Super-Spy variants also exhibited a high level of disorder when no substrate is bound (Quan et al., 2014). Thus, increased Spy flexibility due to reduced stability enhanced chaperone function and was accompanied by tighter substrate binding (Figure 2D; Quan et al., 2014). These findings are another interesting example of destabilizing mutations proving beneficial, as observed by Gong et al. in the evolution of influenza nucleoprotein, whereby stabilizing mutations compensated for otherwise destabilizing mutations that were actually advantageous (Gong et al., 2013). It would be intriguing to determine whether mutations can be isolated that increase the stability of super-Spy variants without affecting their enhanced chaperone activity. Super-Spy variants maintained broad chaperone function against diverse substrates and were not more selective for the substrate used for selection (Figure 2D; Quan et al., 2014). Remarkably, Spy homologs in other species possess some of the exact substitutions that yield super-Spy in *E. coli* (Quan et al., 2014). Thus, protein engineering can enhance our understanding of chaperone function at the amino acid level. This work also raises a valuable design principle in chaperone engineering: introducing instability and flexibility into some chaperones can enhance function.

## Re-engineering Hsp104 to disaggregate neurodegenerative disease substrates

**Figure 3:**
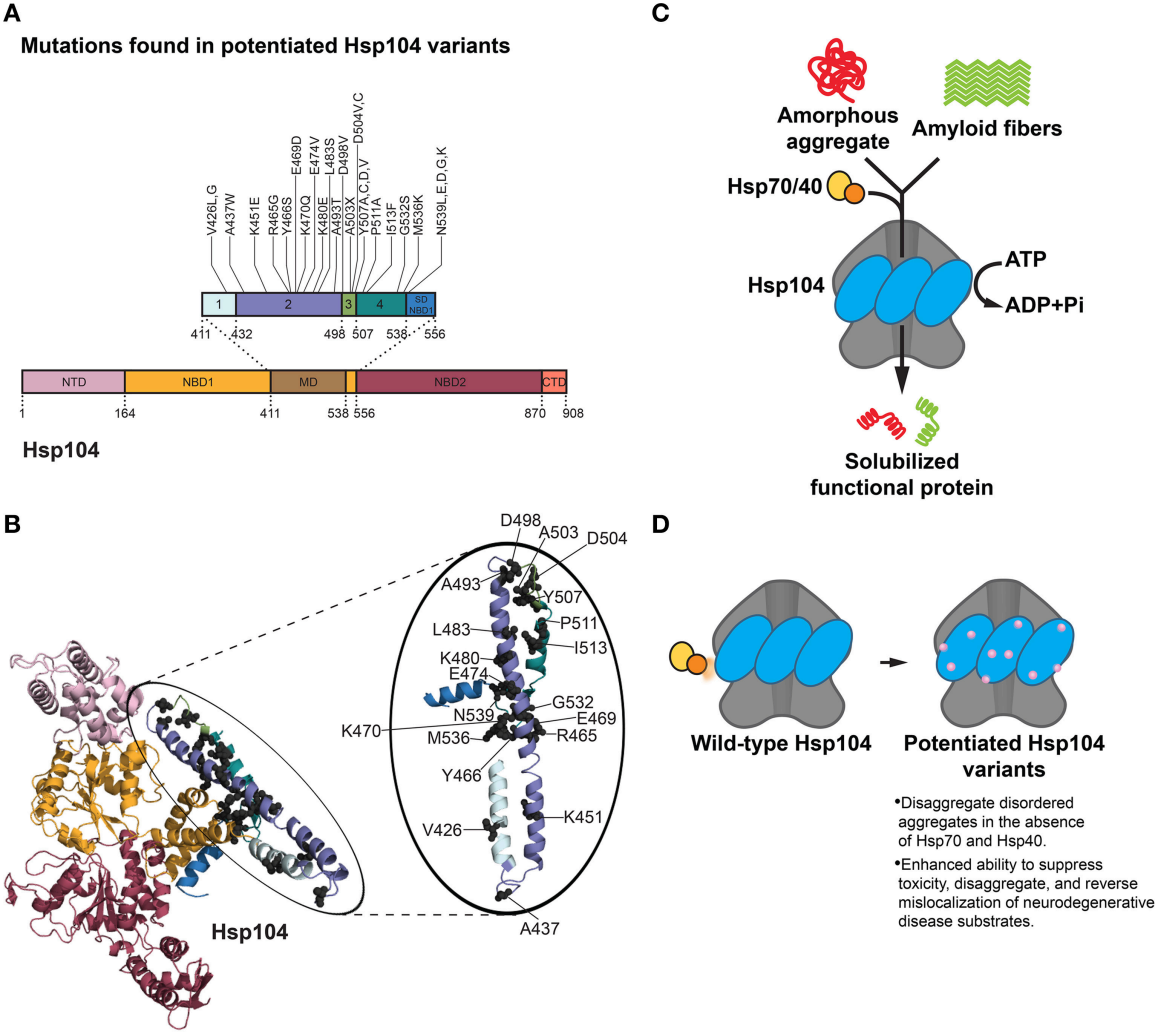
**Hsp104 disaggregation mechanism and features of potentiated variants. (A)** Mutations in potentiated Hsp104 variants surface throughout the middle domain (MD) in helices 1, 2, 3, and 4 and the small domain of nucleotide-binding domain 1 (SD NBD1) (Jackrel et al., 2014, 2015). Interestingly, substitution of A503 with any residue except proline (A503X) yielded a potentiated Hsp104 variant. Several other positions (V426, D504, Y507, and N539) can be substituted for various residues and achieve potentiating effects. Based on Figure 1C in Jackrel et al. (2015). **(B)** Homology model of an Hsp104 monomer (inset: Hsp104 MD) showing the positions of mutations (gray spheres) in potentiated Hsp104 variants. Colors correspond to **(A)**. NTD is from PDB ID: 1KHY and NBD1, MD, and NBD2 are from PDB ID: 1QVR (Lee et al., 2003; Li and Sha, 2003). **(C)** Hsp104 disaggregation mechanism. In collaboration with Hsp70 and Hsp40, Hsp104 unfolds and threads substrate through the central channel of the hexamer using energy from ATP hydrolysis (DeSantis and Shorter, 2012). **(D)** Features of potentiated Hsp104 variants. Potentiated Hsp104 variants disaggregate disordered aggregates in the absence of Hsp70 and Hsp40, and possess an enhanced ability to suppress toxicity, disaggregate, and reverse mislocalization of neurodegenerative disease substrates.

Hsp104 is a hexameric AAA+ ATPase and protein disaggregase found in *Saccharomyces cerevisiae* (DeSantis and Shorter, 2012; Sweeny and Shorter, 2015). Structurally, six Hsp104 protomers form a ring-shaped hexamer with a central channel through which substrate is partially or completely threaded (Parsell et al., 1994; Lum et al., 2004, 2008; Haslberger et al., 2008; Tessarz et al., 2008; Castellano et al., 2015; Sweeny and Shorter, 2015; Sweeny et al., 2015). Each individual Hsp104 protomer is comprised of an N-terminal domain, two nucleotide-binding domains (NBDs) that bind and hydrolyze ATP, a coiled-coil middle domain (MD) that enables communication between domains, and a C-terminal domain necessary for hexamer formation (Figures 3A,B; Sweeny and Shorter, 2015).

Hsp104 possesses an unusual ability to rapidly resolubilize toxic prefibrillar oligomers and amyloid (Shorter and Lindquist, 2004, 2006; Lo Bianco et al., 2008; Shorter, 2008; Vashist et al., 2010; DeSantis et al., 2012; Klaips et al., 2014; Sweeny and Shorter, 2015). Hsp104 couples ATP hydrolysis to the refolding and reactivation of aggregated proteins, and does so optimally in combination with chaperones Hsp110, Hsp70 and Hsp40 (Figure 3C; Glover and Lindquist, 1998; Shorter and Lindquist, 2008; Shorter, 2011; Rampelt et al., 2012). Hsp104 plays a critical role in the construction and deconstruction of various yeast prions (Sweeny and Shorter, 2015; Sweeny et al., 2015), which can confer selective advantages (Shorter and Lindquist, 2005; Newby and Lindquist, 2013). Interestingly, Hsp104 is highly conserved in eubacteria, fungi, protozoa, algae, and plants, yet is absent from metazoa (Vashist et al., 2010; Erives and Fassler, 2015).

Hsp104 represents an interesting potential therapeutic modality for human neurodegenerative diseases rooted in protein misfolding. Hsp104 eradicates preformed fibrils formed by an array of neurodegenerative disease substrates *in vitro*, including tau, polyglutamine, Aβ42, α-synuclein, and PrP (Lo Bianco et al., 2008; Liu et al., 2011; DeSantis et al., 2012). Despite this intrinsic ability, substantial levels of Hsp104 are necessary to achieve only modest disaggregase activity (DeSantis et al., 2012). With such room for improvement, Hsp104 is poised as an ideal disaggregase to re-engineer for targeting the toxic misfolded species underlying multiple neurodegenerative diseases (Jackrel and Shorter, 2014b, 2015). As the structural details of Hsp104 are not yet well understood, it is not an ideal protein for a rational design approach. However, an unbiased approach has been pursued. Thus, a library of Hsp104 MD variants has been screened in an effort to isolate Hsp104 variants that suppress toxicity of aggregated α-synuclein (which is connected to PD), FUS, and TDP-43 (which are connected to ALS and FTD) in yeast (Jackrel and Shorter, 2014a; Jackrel et al., 2014, 2015). The MD of Hsp104 was selected for error-prone PCR mutagenesis as it is a regulatory domain imperative for inter-domain communication between NBD1 and NBD2, and collaboration with Hsp70 (Cashikar et al., 2002; Schirmer et al., 2004; DeSantis and Shorter, 2012; Lee et al., 2013; DeSantis et al., 2014; Sweeny and Shorter, 2015). Limiting library generation to the MD was advantageous in terms of achieving more profound coverage. Coupling this library to a powerful screen against α-synuclein, FUS, and TDP-43 in Δ*hsp104* yeast ultimately led to the isolation of numerous variants that showed impressive rescue of disease substrate toxicity (Figures 3A,B,D; Jackrel and Shorter, 2014a; Jackrel et al., 2014, 2015).

Although the screen yielded an assortment of potentiated variants at particular residues in the MD (and also the small domain of NBD1), the A503 position in helix 3 of the MD surfaced as exceptionally interesting (Jackrel et al., 2014). Substitution of A503 with any residue except proline yielded Hsp104 variants that rescue α-synuclein, FUS, and TDP-43 toxicity in yeast (Figures 3A,B; Jackrel et al., 2014). For example, Hsp104^A503V^ was highly potentiated in its ability to eliminate protein aggregates, suppress toxicity of disease substrates, and correct protein mislocalization in yeast (Gokhale et al., 2005; Jackrel et al., 2014; Torrente et al., 2016). Remarkably, ATPase activity at either NBD1 or NBD2 was sufficient for this potentiated activity (Torrente et al., 2016). However, inhibition of ATPase activity at both NBDs or mutation of key substrate-binding, pore loop tyrosines to alanine in either NBD abolished activity (Torrente et al., 2016). It was determined that the remarkable gain of function in Hsp104^A503V^ was not derived from lower expression of the disease substrates, nor increased expression of Hsp104^A503V^, and was not dependent on the unfolded protein response, heat shock response, or autophagy (Jackrel et al., 2014). Curiously, however, Hsp104^A503V^ confers a growth defect in yeast at 37°C (Schirmer et al., 2004; Jackrel et al., 2014). By contrast, Hsp104^A503S^ was less toxic to yeast at 37°C and protected dopaminergic neurons from α-synuclein-induced neurodegeneration in a transgenic *C. elegans* model of PD (Jackrel et al., 2014). Furthermore, potentiated Hsp104 variants rescued aggregation and toxicity of a wide range of disease-associated FUS, TDP-43 and α-synuclein mutants in yeast (Jackrel and Shorter, 2014a). These enhanced Hsp104 variants also rescued aggregation and toxicity of TAF15, an RNA-binding protein (RBP) with a prion-like domain (PrLD) that is linked to ALS and FTD (Couthouis et al., 2011; King et al., 2012; Li et al., 2013; Jackrel and Shorter, 2014a). Interestingly, potentiated Hsp104 variants were unable to rescue the aggregation or toxicity of EWSR1, another RBP with a PrLD involved in ALS and FTD, which suggests some degree of substrate selectivity (Couthouis et al., 2012; Jackrel and Shorter, 2014a).

The finding that almost any residue substitution at the A503 position resulted in an enhanced Hsp104 variant is intriguing, and suggests that loss of amino acid identity at select positions in the MD rather than specific mutation underpins potentiation (Jackrel et al., 2014; Jackrel and Shorter, 2015). Whether the same is true at other positions in the MD is uncertain, but certainly several positions (e.g., V426, Y507) can be changed to multiple amino acids to yield potentiated variants (Figure 3A; Jackrel and Shorter, 2014b, 2015; Jackrel et al., 2014, 2015). Deletion of motif 2, helix 3, or helix 4 of the MD also yielded potentiated Hsp104 variants, whereas deletion of the entire MD or NTD precluded Hsp104 potentiation (Jackrel et al., 2015; Sweeny et al., 2015).

To investigate the mechanism underlying Hsp104 potentiation, several biochemical aspects of the most effective variants were surveyed. Surprisingly, unlike Hsp104, Hsp104^A503V^ disaggregates disordered aggregates in the absence of Hsp70 and Hsp40 (Jackrel et al., 2014, 2015; Torrente et al., 2016). Additionally, Hsp104^A503V^ translocates unfolded substrates more rapidly than Hsp104, and demonstrates enhanced unfoldase, remodeling, and disaggregase activity against a variety of substrates, including α-synuclein, FUS, TDP-43, TAF15, and SEVI fibrils (Figure 3D; Jackrel and Shorter, 2014a; Jackrel et al., 2014; Castellano et al., 2015). Subunits within the Hsp104^A503V^ hexamer also appear to collaborate differently than Hsp104 to promote disaggregation (Jackrel et al., 2014; Torrente et al., 2014, 2016; Jackrel and Shorter, 2015). A nearly universal theme that surfaced in the isolated potentiated Hsp104 variants is an elevation of basal ATPase activity to ~2–4-fold higher than Hsp104 (Jackrel et al., 2014). Interestingly, several of the evolved GroEL variants also exhibited elevated ATPase activity (Wang et al., 2002), indicating that accelerated ATPase activity may be a common mechanism to enhance chaperone or disaggregase activity. However, one potentiated variant, Hsp104^D504C^, did not display greater ATPase activity than Hsp104 (Jackrel et al., 2014). Thus, enhanced ATPase activity is not an absolute requirement for Hsp104 potentiation. Rather, the one characteristic that unifies the potentiated Hsp104 variants is the ability to disaggregate disordered aggregates *and* amyloid in the absence of Hsp70 and Hsp40 (Figure 3D; Jackrel and Shorter, 2014a; Jackrel et al., 2014, 2015; Torrente et al., 2016). This property could be critical as Hsp70 and Hsp40 can become sequestered or inhibited by disease-associated aggregates and toxic soluble oligomers (Auluck et al., 2002; Hinault et al., 2010; Yu et al., 2014).

By harnessing the power of yeast proteinopathy models in the context of screening a domain-specific library, Hsp104 was re-engineered to potently eliminate aggregation and toxicity of a range of misfolded neurodegenerative disease substrates (Jackrel and Shorter, 2014b, 2015). The potentiated Hsp104 variants bring to light an interesting motif in chaperone engineering, i.e., modifications to regulatory domains as opposed to key residues in ATPase domains or substrate-binding domains often lead to notable improvements in activity. In the case of Hsp104, a single mutation in the regulatory MD yielded dramatically enhanced activity and a therapeutic gain of function (Jackrel and Shorter, 2014b, 2015; Jackrel et al., 2015). We hypothesize that potentiating mutations in the MD relieve its autoinhibitory function or imitate an allosteric event, e.g., Hsp70 binding to the MD (Lee et al., 2013), which activates Hsp104 (Jackrel et al., 2014; Torrente et al., 2016). Additionally, several Hsp104 variants with substitutions at the A503 position appear less stable *in vitro*, and are typically expressed at lower levels *in vivo* (Jackrel and Shorter, 2014a; Jackrel et al., 2014, 2015). In this regard, potentiated Hsp104 variants resemble the super-Spy variants (Quan et al., 2014) in that increased protein instability correlates with enhanced function.

Although our potentiated Hsp104 variants suppress the toxicity of various misfolded disease substrates (Jackrel and Shorter, 2014b, 2015), this enhanced activity would be best honed against a specific disease substrate. Fine-tuning hyperactive Hsp104 variants to improve substrate specificity would be ideal to avoid the nonspecific unfolding of proteins or other off-target effects. These nonspecific effects likely underpin the toxicity of potentiated Hsp104 variants to yeast at 37°C (Jackrel and Shorter, 2014a; Jackrel et al., 2014). Indeed, work by Sauer and colleagues provides insight into how to alter the substrate specificity of a different AAA+ ATPase, ClpX (Farrell et al., 2007). ClpX unfolds and translocates substrates into the chambered protease ClpP, and targets client proteins for degradation through the identification and binding of specific peptide tags (Farrell et al., 2007). ClpX contains a conserved RKH sequence found in pore loops that engages specific degradation tags on clients (Farrell et al., 2007). Single mutations in this RKH sequence greatly alter the substrate specificity of ClpX (Farrell et al., 2007). Specifically, changing RKH to AKH decreased degradation of substrates harboring an ssrA tag, yet improved the degradation of substrates containing three other types of peptide degradation tags (Farrell et al., 2007). This change to the pore loop sequence resulted in a ~300-fold alteration in substrate specificity, demonstrating the critical role of the RKH pore loops in terms of ClpXP substrate recognition (Farrell et al., 2007). A similar tuning of the conserved substrate-binding pore loops (Sweeny and Shorter, 2015) in our potentiated Hsp104 variants for particular clients could prove useful in targeting precise disease substrates, and ultimately provide deeper insight into the underlying mechanisms by which this disaggregase recognizes substrates.

## Directed evolution of enhanced DnaK variants

Hsp70 family members comprise a diverse group of chaperones that have widespread functions in protein folding. Hsp70 assumes a critical role in helping to refold aggregated or misfolded protein as well as in the folding of newly synthesized proteins, among other functions (Mayer and Bukau, 2005; Torrente and Shorter, 2013; Clerico et al., 2015). As such, Hsp70 is a crucial component in protein quality control systems, and specialized forms interact with a large continuum of substrates (Mayer and Bukau, 2005; Clerico et al., 2015). Hsp70 client proteins usually contain hydrophobic stretches, and Hsp70 works cooperatively with its obligate co-chaperone and J-domain containing, Hsp40 (Hartl and Hayer-Hartl, 2002; Genevaux et al., 2007), as well as various nucleotide exchange factors (NEFs) (Rampelt et al., 2011).

**Figure 4:**
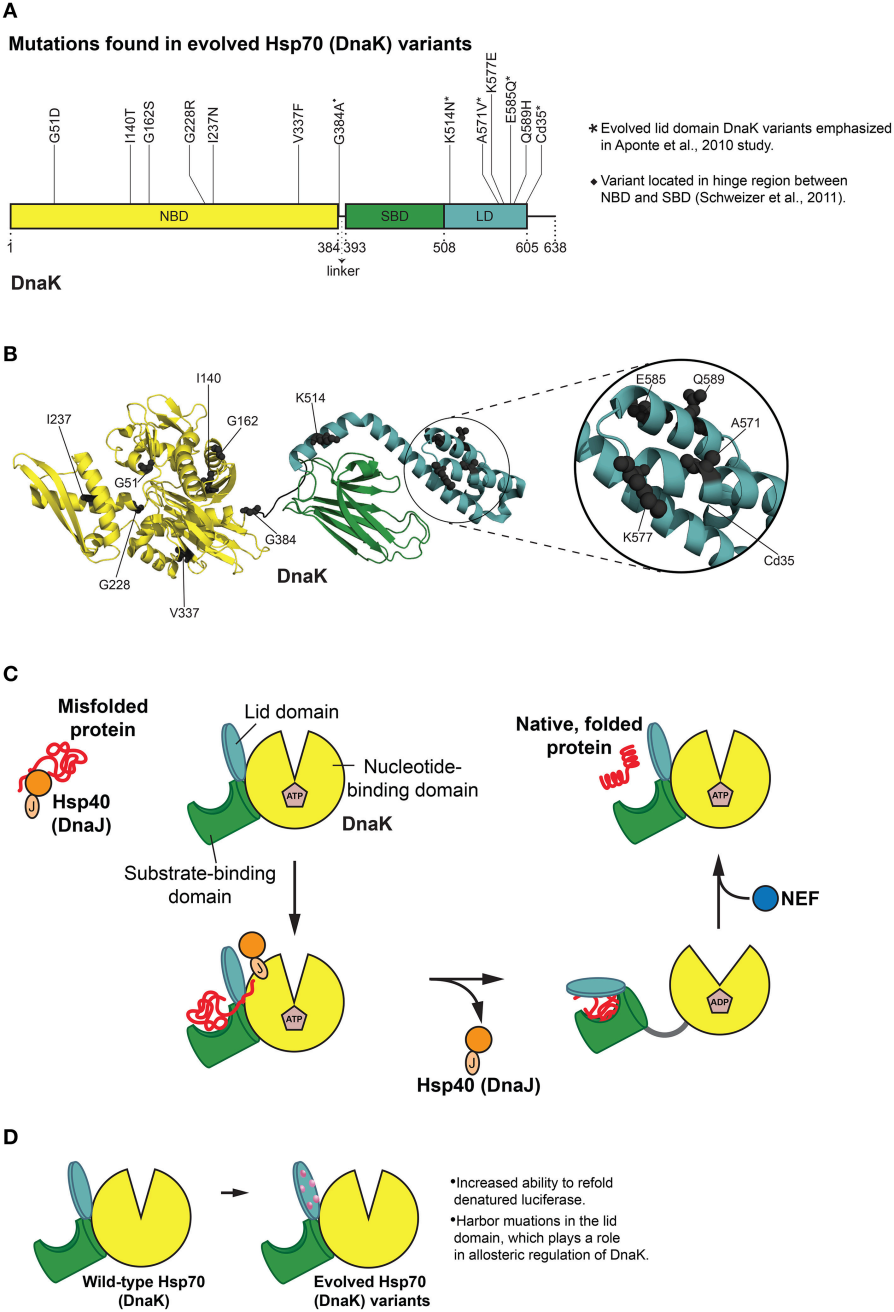
**Hsp70 (DnaK) protein folding cycle and features of evolved variants. (A)** Mutations in evolved DnaK variants localize to the nucleotide binding domain (NBD) and lid domain (LD) of DnaK, rather than the substrate binding domain (SBD; Aponte et al., 2010). Interestingly, one enhanced variant harbors a C-terminal truncation of 35 amino acids (Cd35; Aponte et al., 2010). (^*^) Denotes evolved lid domain DnaK variants emphasized in Aponte et al. (2010) study. (♦) Denotes G384A mutation isolated in subsequent evolution of Cd35 DnaK variant (Schweizer et al., 2011). Based on Figure 2 in Aponte et al. (2010). **(B)** Structure (PDB ID: 2KHO) of ADP-bound DnaK with a 33 residue C-terminal truncation (Bertelsen et al., 2009) showing the positions of mutations (gray spheres) in evolved DnaK variants. Colors correspond to **(A)**. Based on Figure 2 in Aponte et al. (2010). **(C)** DnaK protein folding cycle. Hsp40 (DnaJ) binds and presents substrate to DnaK. DnaK then weakly interacts with substrate in proximity to DnaJ, and DnaJ stimulates ATPase activity of DnaK thus stabilizing substrate binding. A nucleotide exchange factor (NEF) promotes nucleotide exchange, Hsp70 returns to the ATP-bound state and substrate is released (Goloubinoff and De Los Rios, 2007; Kim et al., 2013). **(D)** Features of evolved DnaK variants. Evolved DnaK variants show increased ability to refold denatured luciferase, and contain mutations in the lid domain, which plays a role in allosteric regulation of DnaK (Jiang et al., 2005; Vogel et al., 2006; Swain et al., 2007; Aponte et al., 2010).

Structurally, Hsp70 is comprised of two major domains: an N-terminal nucleotide-binding domain (NBD) and a C-terminal substrate-binding domain (SBD) (Figures 4A,B; Kityk et al., 2012; Clerico et al., 2015). The NBD contains four α-β subdomains (IA, IB, IIA, and IIB) split into two sections by an ATP binding site essential for the ATPase activity needed to power Hsp70 as well as regulating affinity for substrates (Kityk et al., 2012; Qi et al., 2013). Client proteins bind to a pocket in the SBD in the β-sheet subdomain (β-SBD) (Kityk et al., 2012). The SBD also encompasses an α-helical subdomain, termed the lid domain (LD), which acts analogously to a lid that opens and closes to allow for substrate binding and release (Figures 4A,B; Mayer and Bukau, 2005; Kityk et al., 2012). In the absence of any nucleotide or in the presence of ADP, the two domains share little contact and do not act as a functional unit, but when ATP is bound, there is strong allosteric coupling between the two (Qi et al., 2013). Importantly, when ATP binds Hsp70, the affinity for substrate in the SBD is substantially reduced (Landry et al., 1992; Qi et al., 2013). Nonetheless, Hsp70 initially engages substrates in an ATP-bound “open” state and typically recognizes short peptide segments (~7 residues) that are inappropriately exposed in unfolded proteins (Figure 4C; Landry et al., 1992; Misselwitz et al., 1998; Mayer and Bukau, 2005; Clerico et al., 2015). The N-terminal J domain of Hsp40 and substrate binding stimulate ATP hydrolysis by Hsp70, which induces a conformation change in Hsp70 such that substrate peptides are now tightly clamped in an extended form through a groove in the Hsp70 SBD (Figure 4C; Landry et al., 1992; Misselwitz et al., 1998; Qi et al., 2013; Clerico et al., 2015). Hsp70 NEFs then promote exchange of ADP for ATP causing Hsp70 to switch back to a lower affinity state and substrate is released (Figure 4C; Mayer and Bukau, 2005). This complex allosteric control over Hsp70 is vital to the overall activity of the chaperone. Indeed, the effective affinity of Hsp70 for substrates is significantly higher when Hsp70 runs through this cycle (De Los Rios and Barducci, 2014). Intriguingly, the large conformational changes of Hsp70 during its ATPase cycle are not invariably coupled to changes in the overall conformation of the bound substrate protein (Sekhar et al., 2015). Thus, unfolded substrates are likely protected from aggregation while bound to Hsp70 and can explore secondary structural propensities inherent to the polypeptide chain (Sekhar et al., 2015). Hsp70 might also bias folding trajectories of bound substrates as a secondary amide peptide bond cis-trans isomerase (Schiene-Fischer et al., 2002; Swain and Gierasch, 2002). Conversely, Hsp70 also possesses a potent unfoldase activity, which might unfold misfolded structures and reset the energy landscape thereby providing another opportunity for the client to fold correctly (Sharma et al., 2010; Clerico et al., 2015).

Of particular relevance to chaperone engineering is the *E. coli* Hsp70 homolog, DnaK, which associates with ~5–18% of newly synthesized proteins (Bukau et al., 2000). Although both random mutagenesis and rational design methods (O'brien et al., 1996; Montgomery et al., 1999; Hu et al., 2002; Fernández Sáiz et al., 2006) have been employed to explore a range of biochemical properties of DnaK, a more general evolution of this chaperone was not undertaken until quite recently. Reinstein and colleagues utilized a directed evolution approach to isolate DnaK variants that outperformed wild-type DnaK in the refolding of soluble, denatured luciferase (Aponte et al., 2010). A clever *in vivo* selection process and subsequent *in vitro* screen uncovered variants with improved initial refolding rates (Aponte et al., 2010). A selection scheme was used based on bacterial resistance to chloramphenicol (Cm) that was dependent on DnaK function. Bacteria can achieve resistance to Cm by modifying the antibiotic with chloramphenicol acetyl transferase (CAT). Aponte et al. employed a C-terminally truncated CAT variant (CAT_Cd9), which has reduced ability to fold properly and weakens bacterial resistance to Cm (Aponte et al., 2010). Overexpression of DnaK and DnaJ resulted in a rescue of this phenotype, thus allowing cells to grow in Cm (Aponte et al., 2010). This *in vivo* selection system was then exploited to screen *dnaK* libraries for improved rescue of CAT_Cd9 folding under Cm selective pressure (Aponte et al., 2010). In this way, ten enhanced DnaK variants were isolated from the *in vivo* selection and subsequent *in vitro* screen, and two additional enhanced DnaK variants were found in a variation of this procedure (Figures 4A,B; Aponte et al., 2010).

Interestingly, none of the enhanced DnaK variants had mutations in the SBD (Aponte et al., 2010). Instead, half of the substitutions were located in the LD, and the other half in the NBD (Figures 4A,B; Aponte et al., 2010). Mutations in the NBD resided in several different lobes as well as the nucleotide-binding region, while those in the LD resided on every helix with the exception of helix B (Aponte et al., 2010). One variant stood out from the others with single mutations in that it harbors a C-terminal truncation of 35 amino acids (Cd35), thereby eliminating the flexible C-terminal tail of DnaK (Figures 4A,B; Aponte et al., 2010). To gauge the overall chaperone function of the isolated DnaK variants, each variant was purified and screened for the ability to aid in refolding chemically denatured luciferase *in vitro* (Aponte et al., 2010). Relative to wild-type DnaK, four variants displayed greater ability to refold luciferase (in collaboration with co-chaperones) as measured by greater maximal luminescence values, with three of these variants exhibiting increased initial refolding rates (Aponte et al., 2010). Each of the enhanced DnaK variants contained substitutions in the LD, and interestingly, the Cd35 variant displayed the greatest increase in chaperone function (Figures 4A,B; Aponte et al., 2010).

The enhanced activity of Cd35 is surprising as subsequent studies indicate that the C-terminal region of DnaK improves *in vivo* chaperone function, and enhances the *in vitro* protein refolding ability of DnaK (Smock et al., 2011). It was proposed that the C-terminal region interacts with substrate proteins in a transient fashion, as it contains a disordered region that may act as a “flexible tether” between a conserved area of the tail and the SBD (Smock et al., 2011). The differences observed in DnaK function when the C-terminal region is deleted could possibly stem from His-tagged vs. untagged DnaK being used in these studies (Aponte et al., 2010; Smock et al., 2011). The Cd35 variant was further investigated in a subsequent study in which Reinstein and colleagues used a directed evolution approach to further improve chaperone function of the truncated mutant (Schweizer et al., 2011). Error-prone PCR of the Cd35 variant, followed by selection through the aforementioned CAT_Cd9 selection scheme (Aponte et al., 2010), resulted in a variant with a G384A amino acid substitution located in a “hinge position” that connects the NBD and SBD of DnaK (Figures 4A,B; Schweizer et al., 2011). The G384A/Cd35 double mutant displayed enhanced *in vitro* refolding-assistance of denatured firefly luciferase, yet was not as effective as the Cd35 variant alone in this measure of chaperone activity (Schweizer et al., 2011). Further biochemical analysis revealed that the G384A/Cd35 double mutant is similar to the single G384A variant, which is slow to release substrate peptides upon ATP binding (Schweizer et al., 2011). The G384A/Cd35 double mutant is also similar to the Cd35 mutant as it demonstrates faster peptide binding when ADP is bound. However, these changes do not appear to act synergistically in improving luciferase-refolding activity (Schweizer et al., 2011). This result suggests the G384A/Cd35 double mutant is perhaps more selective in folding the CAT_Cd9 substrate, as the combination of mutations offers no enhancement in re-folding the model substrate firefly luciferase. It is interesting that the G384A/Cd35 double mutant appears to have altered substrate specificity, and would be intriguing to test this mutant against a gamut of other substrates or use this mutant as a starting scaffold for evolution of more substrate-specific DnaK variants.

Unlike potentiated Hsp104 variants (Jackrel et al., 2014), the activated DnaK variants exhibited no significant change in intrinsic ATPase activity compared to wild-type DnaK (Aponte et al., 2010). Moreover, co-chaperone (DnaJ and GrpE) stimulation of ATPase activity in the presence and absence of denatured luciferase was very similar to that of wild-type DnaK, although the Cd35 variant as well as E585Q displayed lesser GrpE stimulation overall, possibly indicating differences in nucleotide exchange involving this NEF (Aponte et al., 2010). Perhaps the most intriguing result from this selection and screening approach was that no substitutions in the SBD of DnaK were obtained (Figures 4A,B). In fact, the improved DnaK variants harbored mutations in the LD, which plays a role in the allosteric regulation of DnaK activity (Figures 4A,B,D; Jiang et al., 2005; Vogel et al., 2006; Swain et al., 2007; Aponte et al., 2010). Reinstein and colleagues suggest that a change in the tuning of the DnaK ATPase cycle is critical for enhanced activity against specific substrates (Aponte et al., 2010). The directed evolution of DnaK highlights the complexity of predicting activating mutations in chaperone engineering. Enhancing substitutions often may surface in domains that are not directly involved in substrate binding, yet exert crucial allosteric effects.

Transmission of allosteric signals in Hsp70 involves crucial interdomain interactions between specific regions of the NBD and SBD (Zhuravleva et al., 2012; Zhuravleva and Gierasch, 2015). NMR spectroscopy revealed that two interfaces are stabilized in different states of Hsp70: in the ATP-bound state, an interaction between the NBD and β-SBD is crucial, whereas when substrate and ADP are present, a stabilizing interaction occurs between the β-SBD and α-helical lid (Zhuravleva et al., 2012; Zhuravleva and Gierasch, 2015). Interestingly, each of these interactions works to stabilize an allosterically active form of Hsp70 (in which both substrate and ATP are bound), and the two forms are engaged in an active “tug-of-war” due to contrasting energetic contributions (Zhuravleva et al., 2012; Zhuravleva and Gierasch, 2015). These two interfaces represent “tunable” landscapes in Hsp70 function, as even slight modification to these interactions exerts major effects on the balance between each Hsp70 conformation (Zhuravleva et al., 2012; Zhuravleva and Gierasch, 2015). As such, a resultant change in chaperone function through a shift in ATPase activity or substrate affinity can likely be realized (Zhuravleva et al., 2012; Zhuravleva and Gierasch, 2015). Intriguingly, one of the lid domain DnaK variants (K514N) with enhanced chaperone activity that was isolated in the selection by Reinstein and colleagues, resided at the β-SBD:α-helical lid interface (Aponte et al., 2010). These separate findings appear to connect the structural basis for allosteric communication in DnaK to a functionally relevant *in vivo* selection of improved chaperone variants. The bridging of key structural findings and Hsp70 functional studies will likely reveal aspects important in effectively engineering augmented forms of this chaperone.

## Selectively increasing Hsp70 affinity for neurodegenerative disease substrates via rational design

Though unbiased screens have proven valuable in uncovering enhanced chaperone variants, rational design strategies are another useful avenue for improving specific chaperone features. Such an approach was employed by Vendruscolo and colleagues to increase the affinity of Hsp70 for several proteins associated with neurodegenerative disease (Aprile et al., 2015). To engineer human Hsp70 to more potently bind and have improved anti-aggregation activity against α-synuclein and Aβ42, Vendruscolo and colleagues used a tactic similar to that pioneered to craft gammabodies for inhibition of fibril assembly (Ladiwala et al., 2012; Perchiacca et al., 2012). A complementary peptide that will specifically bind a target epitope in α-synuclein (the NAC region) or Aβ42 was transplanted onto the C-terminus of Hsp70 (Aprile et al., 2015). The affinity of the grafted Hsp70 variant designed for α-synuclein, termed GHsp70-NAC, for a model substrate was similar to that of wild-type Hsp70, indicating that affinity for other chaperone substrates was unchanged (Aprile et al., 2015). Similarly, GHsp70-NAC refolded chemically denatured luciferase similarly to wild-type Hsp70 (Aprile et al., 2015). Binding experiments using dansyl-α-synuclein showed GHsp70-NAC had increased binding affinity for substrate, and fluorescence competition assays further supported that GHsp70-NAC favorably bound the target region of α-synuclein (Aprile et al., 2015). Furthermore, it was demonstrated that GHsp70-NAC had stronger binding affinity for dansyl-α-synuclein (relative to wild-type Hsp70) in *E. coli* cellular extracts (Aprile et al., 2015).

To rule out the possibility that solely lengthening the C-terminal region was causing the changes in target substrate binding, Vendruscolo and colleagues created an Hsp70 to selectively bind Aβ42 (GHsp70-Aβ) (Aprile et al., 2015). This Hsp70 variant showed increased binding affinity for Aβ42, and an affinity for α-synuclein comparable to that of wild-type Hsp70 (Aprile et al., 2015). Thus, the inclusion of a complementary peptide, and not just the extension of the C-terminal region of Hsp70, results in the enhanced binding of target substrate (Aprile et al., 2015). α-synuclein and Aβ42 aggregation assays revealed that the grafted Hsp70 variants had enhanced aggregation inhibition activity toward their target substrates (Aprile et al., 2015). Overall, grafting a complementary peptide onto Hsp70 designed to bind a particular substrate is an exciting approach that could be easily translated to other chaperones and disaggregases. It would be interesting to use this strategy for tailoring individual chaperones toward specific misfolded proteins involved in neurodegenerative disease.

## Combining functionalities to create activated chimeric folding enzymes

While most molecular chaperone engineering approaches improve specific aspects of an existing protein scaffold, another interesting tactic is to exchange specific chaperone domains to combine different functionalities and yield enhanced chaperone chimeras. This approach was used to improve the chaperone activity of the human prolyl isomerase FKBP12 (hFKBP12) (Geitner and Schmid, 2012). hFKBP12 is a modest catalyst of protein folding reactions limited by proline isomerization, as it only contains a prolyl isomerase active site, and no inherent chaperone domain (Geitner and Schmid, 2012). As prolyl isomerases need chaperone function to properly promote protein folding, Schmid and colleagues inserted three natural chaperone domains into a loop in hFKBP12 to boost folding activity of the enzyme (Geitner and Schmid, 2012). Specifically, the apical domain of GroEL, the chaperone domain of yeast protein disulfide isomerase (PDI) and the chaperone domain of the periplasmic *E. coli* chaperone SurA were used to create chimeras with hFKBP12 (Geitner and Schmid, 2012). Both protein folding and peptide isomerization activity were measured to assess the activity of the chimeras relative to hFKBP12, and although the chimeric proteins showed a general decrease in peptide isomerization activity, they displayed a strong improvement in protein folding ability (Geitner and Schmid, 2012). Overall, the addition of molecular chaperone domains to folding enzymes to create a separation of catalytic and client protein binding is an interesting approach to engineer synthetic folding enzymes with amplified function. It will be interesting to determine whether other multidomain chimeric chaperones can be developed for specific functions.

## Risks of re-engineering molecular chaperones and protein disaggregases

Though enhanced molecular chaperones and disaggregases could be advantageous to the cell (especially in the context of excessive deleterious protein misfolding), it is also possible that inappropriate hyperactivity of these proteins could have detrimental effects under some circumstances. Indeed, the potentiated Hsp104 variant, Hsp104^A503V^, is toxic to yeast under mild thermal stress conditions of 37°C (Schirmer et al., 2004; Jackrel and Shorter, 2014a; Jackrel et al., 2014). We suggest that under these mild stress conditions, key yeast proteins become metastable and are subsequently inappropriately targeted and unfolded by Hsp104^A503V^ (Jackrel and Shorter, 2014a, 2015; Jackrel et al., 2014). This nonspecific unfolding likely results in the observed growth defect, which can be suppressed by overexpression of Hsp90 or Hsp40 (Sis1; Schirmer et al., 2004). Thus, further engineering is warranted to tailor potentiated Hsp104 variants to target specific substrates and prevent off-target effects (Jackrel and Shorter, 2015). Alternatively, transient, inducible bursts of expression of potentiated Hsp104 variants at times when they are most needed might also minimize potentially toxic side effects. It is plausible that these detrimental effects observed in enhanced Hsp104 variants might also occur in engineered molecular chaperones with enhanced activity. Indeed, overexpression of even wild-type molecular chaperones (e.g., Hsp70) at inappropriate times can be detrimental (Feder et al., 1992). Thus, the expression of engineered molecular chaperones may need to be tightly controlled or their activity may need to be fine-tuned to specific targets to minimize unwanted off-target effects. Overall, balancing enhanced activity with minimal unfavorable effects will be a key challenge to overcome. Overcoming this challenge will likely be invaluable for combating toxic misfolding events in various diseases.

## Conclusions and outlook

Molecular chaperones and protein disaggregases are critical components of proteostasis networks. Thus, gaining a greater appreciation for how these complex folding machines function on both structural and mechanistic levels is of substantial importance. Ultimately, understanding how to enhance the activity of molecular chaperones and protein disaggregases may be useful for combating toxic protein misfolding events in neurodegenerative disease, as well as large-scale purification of aggregation-prone proteins for basic and therapeutic purposes. Overall, insights gleaned from the successful engineering endeavors outlined above emphasize a number of crucial themes in developing enhanced chaperone variants. Several mechanisms for enhancing activity have been uncovered, including increasing ATPase activity, altering substrate-binding regions, grafting substrate-specific binding sites onto chaperones, and exchanging specific chaperone domains to create improved chaperone chimeras. Furthermore, introducing flexibility by making beneficial destabilizing mutations into chaperones, and targeting regulatory domains as opposed to substrate-binding domains for modification, have proven valuable strategies for engineering several chaperones.

Looking to the future, it will be extremely interesting to synthesize themes from previous chaperone and disaggregase engineering studies and apply them to re-engineering the TRiC chaperonin (Lopez et al., 2015) or the metazoan protein disaggregase system: Hsp110, Hsp70, and Hsp40 (Torrente and Shorter, 2013; Finka et al., 2015; Nillegoda and Bukau, 2015) to more effectively target detrimental misfolding events connected to neurodegenerative disease. In this regard, several lessons learned from our efforts to potentiate Hsp104 activity are particularly encouraging. At first glance, it might seem improbable or impossible to improve existing chaperone or disaggregase activity, which has been hewn over the course of millions of years of evolution. However, such a view overestimates the optimality of natural proteins for specific tasks. Indeed, we now have hundreds of potentiated Hsp104 variants with improved disaggregase activity against TDP-43, FUS, and α-synuclein misfolding (Jackrel et al., 2014, 2015). Remarkably, some of these potentiating changes were extremely subtle. For example, removal of a methyl group from a side chain or addition or removal of a single methylene bridge yielded potentiated forms of Hsp104 (Jackrel et al., 2014, 2015). The relative ease at which potentiated Hsp104 variants were uncovered leads us to hypothesize that neuroprotection in diverse devastating diseases could be broadly realized via remarkably subtle modifications to existing human chaperones. Moreover, because relatively small changes in primary sequence can yield large increases in chaperone or disaggregase activity (Wang et al., 2002; Aponte et al., 2010; Jackrel et al., 2014; Quan et al., 2014), small molecules that bind in the appropriate regions could also have potentiating effects on wild-type chaperones or disaggregases. Thus, it will be critical to isolate small-molecule enhancers of chaperone or disaggregase activity, which might be more immediately translatable. Initial endeavors in this area are yielding promising results (Wang et al., 2013; Pratt et al., 2015). Developing effective ways to enhance chaperone and disaggregase activity holds promise for a wide spectrum of applications.

## Author contributions

All authors listed, have made substantial, direct and intellectual contribution to the work, and approved it for publication.

### Conflict of interest statement

The authors declare that the research was conducted in the absence of any commercial or financial relationships that could be construed as a potential conflict of interest.
